# Quipazine Elicits Swallowing in the Arterially Perfused Rat Preparation: A Role for Medullary Raphe Nuclei?

**DOI:** 10.3390/ijms21145120

**Published:** 2020-07-20

**Authors:** Victor Bergé-Laval, Christian Gestreau

**Affiliations:** Aix-Marseille Université and INSERM, Institut de Neurosciences des Systèmes, UMR 1106, Bd Jean Moulin, 13005 Marseille, France; victor.berge-laval@etu.univ-amu.fr

**Keywords:** swallow, breathing, central pattern generators, serotonin, neuromodulation

## Abstract

Pharmacological neuromodulation of swallowing may represent a promising therapeutic option to treat dysphagia. Previous studies suggested a serotonergic control of swallowing, but mechanisms remain poorly understood. Here, we investigated the effects of the serotonergic agonist quipazine on swallowing, using the arterially perfused working heart-brainstem (in situ) preparation in rats. Systemic injection of quipazine produced single swallows with motor patterns and swallow-breathing coordination similar to spontaneous swallows, and increased swallow rate with moderate changes in cardiorespiratory functions. Methysergide, a 5-HT2 receptor antagonist, blocked the excitatory effect of quipazine on swallowing, but had no effect on spontaneous swallow rate. Microinjections of quipazine in the nucleus of the solitary tract were without effect. In contrast, similar injections in caudal medullary raphe nuclei increased swallow rate without changes in cardiorespiratory parameters. Thus, quipazine may exert an excitatory effect on raphe neurons via stimulation of 5-HT2A receptors, leading to increased excitability of the swallowing network. In conclusion, we suggest that pharmacological stimulation of swallowing by quipazine in situ represents a valuable model for experimental studies. This work paves the way for future investigations on brainstem serotonergic modulation, and further identification of neural populations and mechanisms involved in swallowing and/or swallow-breathing interaction.

## 1. Introduction

Despite extensive experimental and clinical studies, swallowing remains one of the most important and least appreciated functions [1]. Dysfunctions in the brainstem’s swallowing central pattern generator (swCPG) and other medullary or supra-medullary structures may be an important causal factor of oropharyngeal dysphagia, a severe disease whose neural pathophysiology remains poorly understood [2,3,4].

Neurophysiological investigations have identified the nucleus of the solitary tract (NTS) as a key brainstem structure responsible for the pharyngeal phase of swallowing [5,6], but the exact location of the swCPG is still controversial. In addition, the precise neural mechanisms operated by the swCPG are obscure, and the interactions between the central pattern generator for breathing (rCPG) and the swCPG are not fully characterized [7,8]. This lack of knowledge may be explained by the complexity of brainstem circuitry, the paucity of information on the phenotypes of the swCPG neurons and their mechanisms of neuromodulation. Furthermore, the experimental protocols and/or animal models required to study swallowing and swallow-breathing coordination are generally complex, time-consuming and expensive [8,9,10,11].

In some investigations, the arterially perfused working heart-brainstem (in situ) preparation has been used as an advantageous experimental model to study swallowing and record brainstem neuronal activity [12,13,14,15]. However, few spontaneous swallows occurred in situ [16], and several methods were used to evoke swallowing, such as mechanical stimulation of the pharyngeal cavity [15], manual injection of water into the oral cavity [17] and electrical stimulation of the superior laryngeal nerves [13,14]. To our knowledge, a pharmacological method to enhance swallowing over a long period has not been tested in situ.

Previous in vivo studies identified a precise region in the nucleus of the solitary tract (NTS) where injection of glutamate or glutamatergic agonists acting on N-Methyl-D-Aspartate (NMDA) or non-NMDA receptors (kainate or quisqualate) strongly elicited rhythmic swallows and central apnea [6,18]. This region, also called the “trigger zone” for swallowing, is thought to correspond with glutamatergic terminals from upper airway afferent fibers, forming synapses onto swCPG interneurons [6]. Elicitation of swallowing was also observed after peripheral or central injection of serotonergic agonists [5,19]. Interestingly, compounds such as quipazine (QPZ), a serotonergic agonist with main affinity and selectivity for 5-HT2A receptors, have been suggested to exert a central stimulatory effect at the NTS level [5]. Contrary to glutamate effects, QPZ injection in vivo produced single or “isolated” swallows and increased the swallow rate over a long period [5,19], which represents an optimal condition to study swallow-breathing interaction, and a good alternative method to evoke swallowing [8]. In agreement with the role of bulbar serotonergic control of swallowing [5], mice with central 5-HT deficiency had lower swallow rates than control animals [20]. However, inhibitory effects of serotonergic agonists on swallowing, including QPZ, have also been reported [21,22]. Thus, previous in vivo experiments on serotonergic control of swallowing have led to conflicting data. These opposite effects have been interpreted as resulting from differences in drug concentration and protocols of anesthesia between studies [19].

We hypothesized that QPZ at a dose similar to that used in vivo [5] may stimulate swallowing in the arterially perfused working heart-brainstem preparation of an adult rat (an in situ preparation) which requires no anesthesia [15,23]. The aims of our study were (1) to investigate the effects of QPZ on swallowing and breathing in situ, (2) to compare swallowing motor patterns and swallow-breathing phase relationship between spontaneous and QPZ-induced swallows, and (3) to identify brainstem nuclei mediating the stimulatory effect of QPZ on swallowing. Identifying both the neural structures and neurotransmitters (or neuromodulators) involved in swallowing represents one of the challenges to better understand this important function, and to find therapeutic strategies for patients suffering from swallowing disorders [1,2,3,24,25,26].

## 2. Results

### 2.1. Systemic QPZ Effects on Cardiorespiratory Parameters

Mean group data showed that systemic injection of QPZ (1.5 µM/kg) significantly increased respiratory frequency (Rf) (23 ± 9 vs. 30 ± 17 cycles/min, in control vs. QPZ groups, respectively; *p* < 0.05; Table S1). This effect was not consistent across the preparations since no obvious change (4/16) or decrease in Rf (4/16) was also detected. Changes in Rf were observed 108 ± 36 sec after the injection and lasted for about 18 min (Table S1). The mean increase in Rf was associated with non-significant decreases in both inspiratory and expiratory durations compared to control values (0.78 ± 0.32 vs. 0.76 ± 0.25 sec for inspiration, *p* = 0.72, and 1.98 ± 0.9 vs. 1.74 ± 0.99 for expiration, *p* = 0.48; Table S2). Systemic QPZ significantly increased blood pressure (BP) by 25% ± 12% (87 ± 43 vs. 109 ± 61 mmHg in control vs. QPZ groups, respectively; *p* < 0.001; Table S3). This hypertensive effect was consistent across the preparations, detected 69 ± 27 sec after injection, and lasted up to 30 min post-injection. Systemic QPZ injection had no effect on heart rate (345 ± 51 vs. 341 ± 45 bpm in control vs. QPZ groups, respectively; *p* = 0.73; Table S4).

### 2.2. Characterization of Swallows

**Figure 1:**
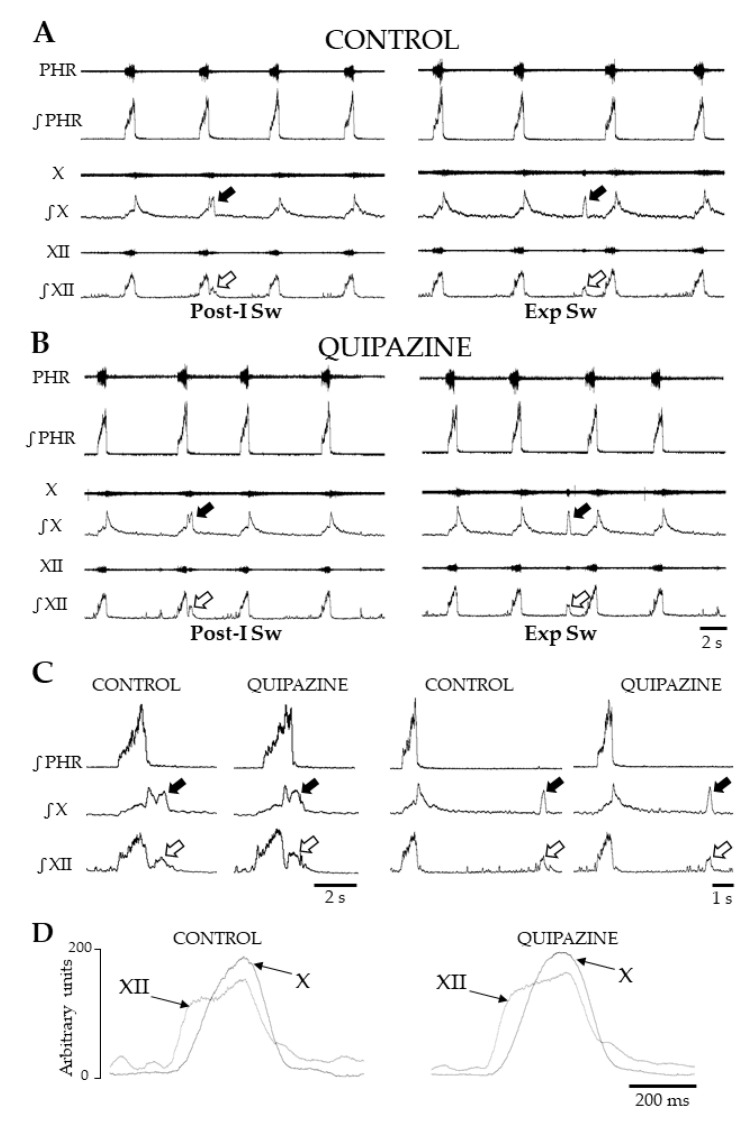
Patterns of breathing and swallowing in situ. Representative traces showing raw and integrated nerve activities during breathing and swallowing in control condition (Control) (**A**) and after systemic quipazine (QPZ; 1.5 µM/kg in aCSF) (**B**). In both cases, single swallows were observed, and two types of swallow were identified. Swallows were characterized by concomitant vagal (X, solid arrow) and hypoglossal (XII, open arrow) bursts, occurring either in early expiration (post-inspiratory swallows, Post-I Sw) or later in expiration (Exp Sw). Enlarged views of single Post-I and Exp Sw in control and QPZ conditions (**C**). Note that Exp Sw were always preceded and followed by central expiration. Averaged envelopes computed from integrated X and XII nerve activities during Exp Sw (*n* = 5) in control and QPZ conditions in the same preparation (**D**). Swallowing-related bursts had similar motor patterns and delays between the starts of the XII and X bursts across conditions. This physiological delay reflects the normal sequence of the pharyngeal phase of swallowing. PHR: phrenic nerve activity.

All rats analyzed in this study had eupneic patterns of breathing at the start of recordings and displayed spontaneous swallows over the entire 15 min control period (Figure 1). These spontaneous events were always isolated within the respiratory cycle, i.e., a single swallow occurred per respiratory cycle (Figure 1A). They were characterized by typical swallowing-related bursts on hypoglossal (XII) and vagal (X) nerves associated with no (or very small) phrenic activity (Figure 1). Swallowing was consistently observed after systemic QPZ injection at a final concentration of 1.5 µM/kg in the artificial cerebrospinal fluid (aCSF) (Figure 1B). Similarly to spontaneous events, all swallows elicited after injection of QPZ were isolated.

In both control and QPZ conditions, two types of swallows were distinguished according to their timing of initiation within the central respiratory cycle. Swallows occurring during the first 20% of the normalized duration of the expiratory phase, i.e., during post-inspiration, were referred to as post-inspiratory swallows (Post-I Sw) (Figure 1A–C). Swallows occurring later in expiration were referred to as expiratory swallows (or Exp Sw) (Figure 1A–D). Swallow duration did not differ between control and QPZ conditions (0.53 ± 0.11 vs. 0.52 ± 0.14, respectively, *p* = 0.43, Table S5). More detailed comparisons of duration, amplitude and area under the curve (AUC) of hypoglossal (XII) and vagal (X) swallowing-related bursts, and delays between the starts of XII and X bursts (Figure 1D), were made across conditions. All parameters did not significantly change between the two groups (Figure S1; Table 1 and Table S5). Therefore, swallows elicited by QPZ had similar motor patterns to spontaneous swallows.

### 2.3. Systemic QPZ Increased Swallow Rate

**Figure 2:**
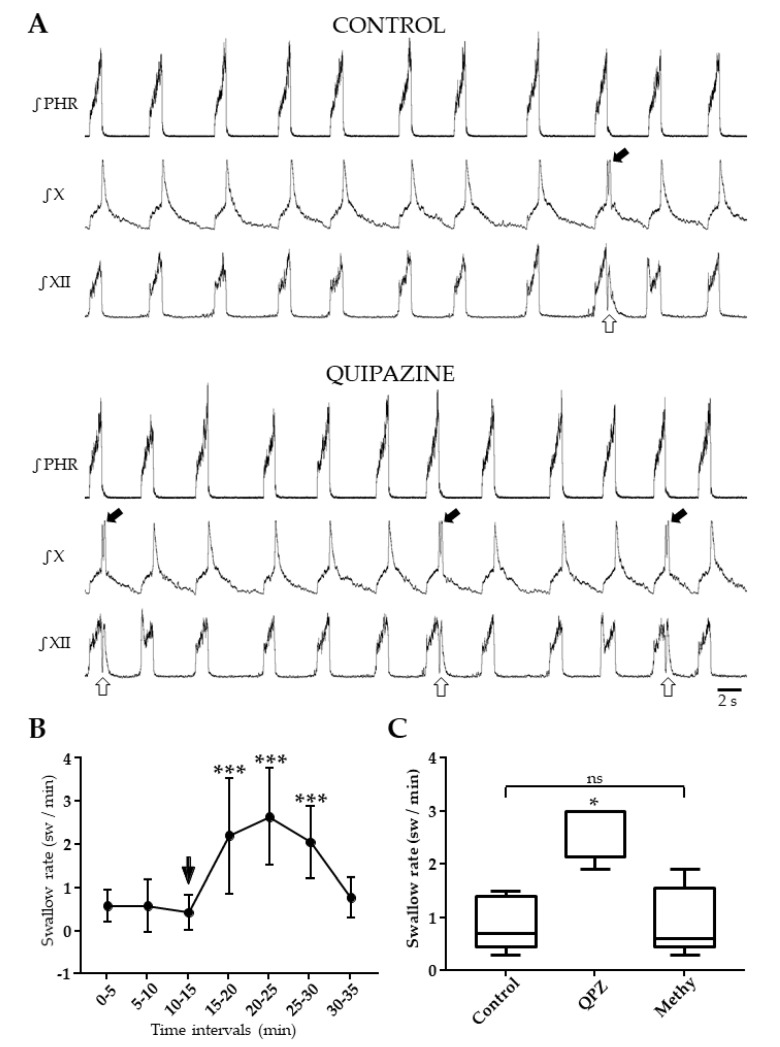
Effect of systemic quipazine (QPZ, 1.5 µM/kg) on swallowing, and its blockade by methysergide (Methy, 1µM/kg). Integrated nerve activities in the same preparation during control and QPZ conditions, showing a drug-induced increase in swallowing (**A**). Swallows were identified by hypoglossal (XII, open arrow) and vagal (X, solid arrow) bursts. Changes in swallow rate, expressed as swallows per min (sw/min) per 5 min intervals, 15 min before (control) and 20 min after QPZ injection (arrow) (**B**). Comparisons of values with a Kruskal–Wallis test indicated the significant effect of QPZ (*p* < 0.001). Post-hoc tests revealed no change in swallow rate over the 15 min control period (*p* = 0.43), and a significant increase in swallow rate for 15 min after drug injection compared to control values (***, *p* < 0.001 for all comparisons). Methysergide reversed the excitatory effect of QPZ on swallowing (**C**). Boxplots representing swallow rates measured for 15 min in control conditions, for 5 min after systemic QPZ injection, and for 15 min after adding Methy into aCSF. A one-way ANOVA showed a significant difference across conditions (F = 22.9, *p* < 0.01). Swallow rate after QPZ injection was significantly increased compared to both control and Methy conditions (*, *p* < 0.05). Swallow rate measured after Methy injection did not differ from control values (*p* = 0.87, ns: not significant).

Spontaneous swallows were observed at slow rate (0.5 ± 0.4 sw/min) without significant changes over the 15 min control period (Figure 2, Table S6). Swallow rate (SR) was significantly increased 105 ± 28 sec after QPZ injection, with a peak effect around 5–6 min following drug injection (Figure 2A, Table S6). This increase lasted for 15 min, and the mean SR over this period was 2.3 ± 0.8 sw/min (*p* < 0.001; Table S6). The magnitude of increase in SR was variable across the preparations, as reflected by standard deviation values in Figure 2A. This was also revealed in the individual (range 1.1 to 28) or averaged (8.5 ± 7.6) means ratio, another index of the magnitude of change in SR across conditions (Table S7). We further determined if this variability of responses to QPZ could be attributable to changes in Rf and/or BP measured in the same preparations. This was not the case, as revealed by the lack of significant correlation between the magnitude of increase in SR and changes in either Rf (*r* = 0.358; *p* = 0.20) or BP (*r* = 0.519; *p* = 0.06; Table S7). In other experiments (*n* = 5), spontaneous swallows were first recorded for 15 min (control period), then QPZ (1.5 µM/kg) was added to aCSF and SR was measured for 5 min. Finally, the 5-HT2 receptor antagonist methysergide (Methy, 1 µM/kg) was also added to aCSF and SR was measured for 15 additional min. Multiple comparisons showed a significant difference between the three conditions (*p* < 0.05; Figure 2B; Table S8). A significant increase in SR was found in preparations injected with QPZ alone (*p* < 0.05 for both comparisons), whereas SR did not change between control and Methy conditions (*p* = 0.99; Figure 2B; Table S8). Therefore, the stimulatory effect seen with QPZ alone was reversed by methysergide. In three additional rats, SR was measured for 15 min before and after injection of Methy alone (1µM/kg). Results revealed that the 5-HT2 receptor antagonist methysergide per se had no effect on swallowing (0.79 ± 0.34 vs. 0.66 ± 0.29 sw/min in control and drug conditions. respectively; *p* = 0.51; Table S9).

### 2.4. Analyses of Swallow-Breathing Coordination and Effect of Swallowing on Central Respiration

**Figure 3:**
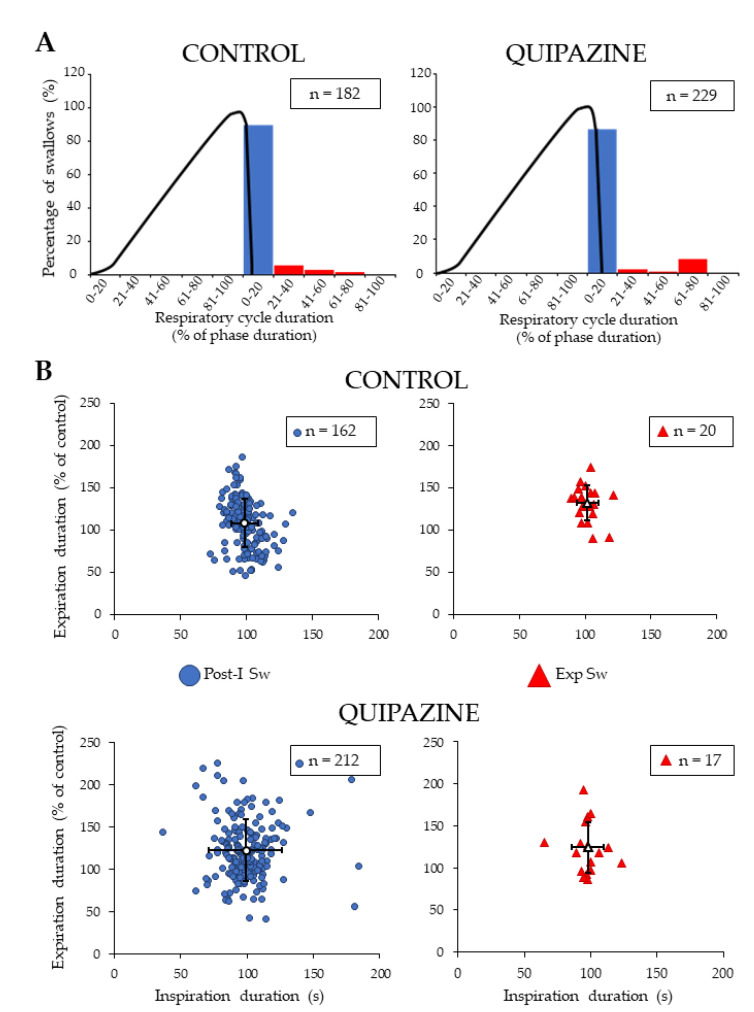
Analyses of swallow-breathing relationship. Occurrence of single swallows within the normalized swallow-breathing cycle in control and quipazine (1.5 µM/kg) groups (**A**). Histograms represent the percentage of swallows observed within the inspiratory and expiratory phases of the swallow-breathing cycles. The black line corresponds to the averaged integrated phrenic activity during fictive breathing and thus delineate the central inspiratory phase. Note that all of the swallows occurred in expiration, most of them being observed in post-inspiration (blue symbols). Effects of swallowing on central breathing (**B**). For both post-inspiratory (Post-I Sw) and expiratory (Exp Sw, red symbols) swallows (numbers indicated in boxes), duration of inspiration and expiration of the swallow-breathing cycle was normalized and compared to control breathing cycles. There was no significant change in inspiration or expiration phase duration induced by Post-I or Exp swallows in either control (upper panels) or quipazine (lower panels) conditions.

Analyses of the phase-relationship between swallowing and breathing were performed using single spontaneous swallows (*n* = 182) and QPZ-induced swallows (*n* = 229) (Figure 3A). In both cases, no swallowing occurred during central inspiration. Most of the swallows (~85%) corresponded to Post-I Sw; Exp Sw were also observed (Figure 3A). Of note, none of the Exp Sw occurred at the expiratory to inspiratory phase transition, as attested by the lack of events in the last percentile (81%–100%) of the normalized expiratory phase duration of the swallow-breathing cycle (Figure 3A). Thus, each Exp Sw was preceded and followed by expiration, as shown in the electrophysiological traces (Figure 1A–D). Distribution of Post-I and Exp Sw within the respiratory cycle did not change across conditions (Χ2 (1) = 0.79, *p* = 0.38). In vivo, Post-I Sw prematurely terminated the preceding phrenic burst without affecting the central expiratory phase, whereas Exp Sw prolonged expiration without affecting the central inspiratory phase [8,27]. To perform similar analyses of the relationship between swallowing and central respiration in situ, we measured the effects of Post-I and Exp Sw on the inspiratory and expiratory phases of swallow-breathing cycles across conditions. Contrary to the studies mentioned above, Post-I or Exp Sw did not significantly change the duration of inspiration or expiration in situ, whether the swallows were spontaneous or induced by QPZ (Figure 3B).

### 2.5. Effects of Central Drug Injections on Swallowing

**Figure 4:**
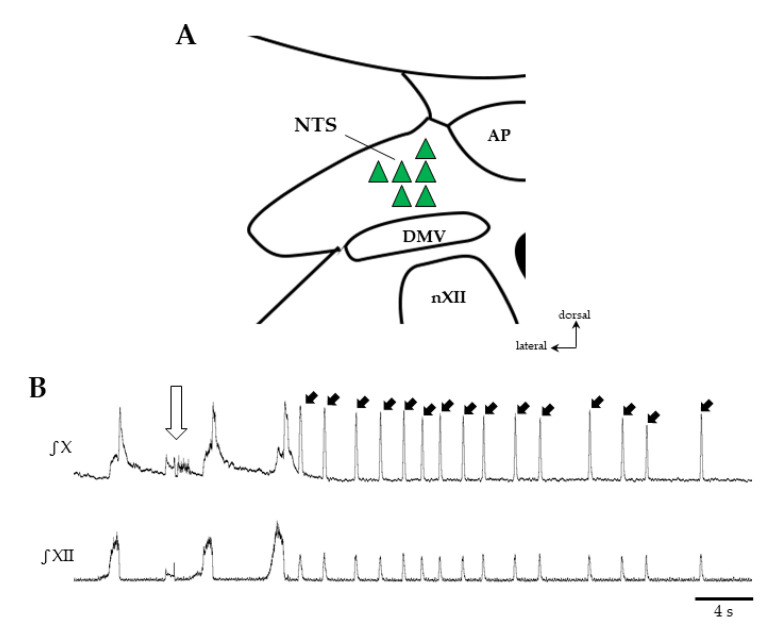
Effects of central drug microinjections in the nucleus of the solitary tract (NTS) on swallowing. Sites of drug injection within the NTS (**A**). Microinjection of N-Methyl-D-Aspartate (NMDA, 1 mMol, 10–50 nL) in the NTS (green triangles) elicited swallowing, whereas quipazine (QPZ 3 mM, 50–200 nL) injected at the same sites did not. Representative traces of vagal (X) and hypoglossal (XII) nerve discharges after NMDA injection in NTS (open arrow) showing repetitive swallows (solid arrows pointing to the X burst) and central apnea (**B**). DMV, dorsal motor nucleus of the vagus nerve; AP, area postrema; nXII, motor nucleus of the hypoglossal nerve.

Microinjection experiments first targeted the NTS (*n* = 6), a key structure involved in swallowing which also has a likely role in mediating the effects of serotonin [5,19]. To identify the swallowing trigger zone within the NTS, N-Methyl D Aspartate (NMDA, 1 mMol, 10–50 nL) was first injected (Figure 4). Then, the pipette containing the drug was removed and NMDA was replaced by QPZ (3mM, 50–200nL). After repositioning of the pipette at the same coordinates, QPZ was injected into the NTS (*n* = 6; Figure 4A). All NMDA microinjections resulted in repetitive swallows and central apnea (Figure 4B). In contrast, no swallow could be observed when QPZ was microinjected in the NTS swallowing trigger zone. This remained true even after repeated injections (two times) of the highest volume (200 nL) a minute apart, or after increasing drug concentration to 5 mM.

**Figure 5:**
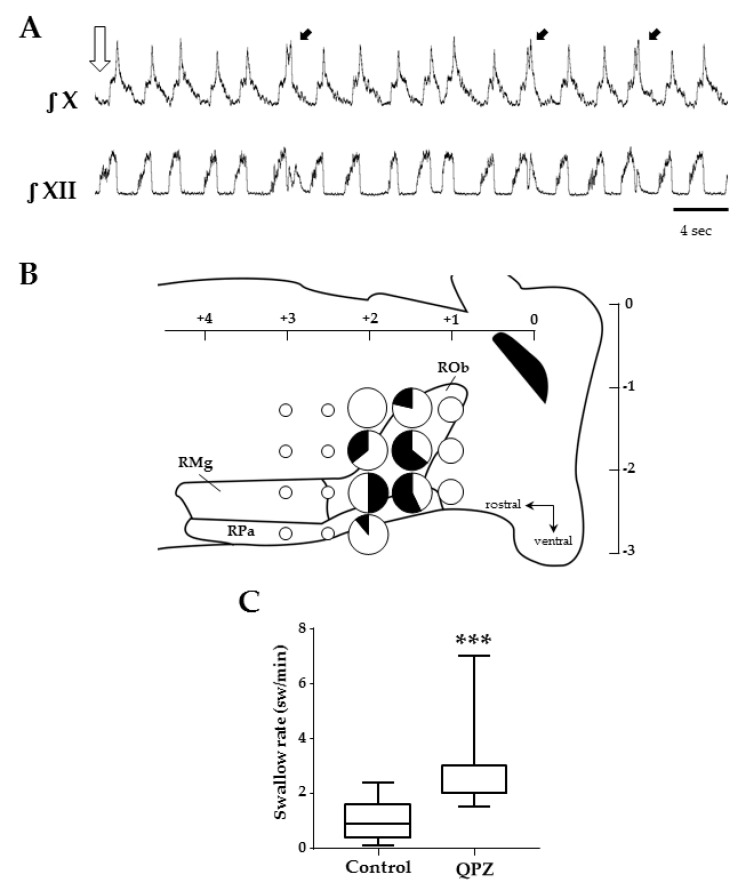
Effects of quipazine microinjections in the medullary raphe nuclei on swallowing. Example of swallows (black arrows) elicited after QPZ injection (open arrow) in the caudal medullary raphe (+1.5 mm from obex, −1.75 mm below the surface) (**A**). Schematic representation of the raphe medullary nuclei, and the 18 sites tested in 15 preparations to map the effects of QPZ on swallowing (**B**). Sites are represented as pie charts with different sizes, according to the total number of injections made in different preparations (small, medium and big sizes corresponding to 1–2, 3–7 and 8–14 injections, respectively), and different colors depending on the effect of QPZ (white: no change; black: increase in swallow rate). Mixed color pie charts indicate sites where QPZ produced both effects and display percentages of the observed effects. Note that 3 adjacent sites were identified in caudal parts of the raphe pallidus and obscurus where 50% or more of the tested injections elicited an increase in swallow rate. Swallow rates in control conditions and after successful QPZ injections in the raphe nuclei (**C**). Note that QPZ significantly increased the swallow rate compared to control values (***, *p* < 0.001). ROb, RPa and RMg: raphe obscurus, pallidus and magnus, respectively.

In other experiments, QPZ (3mM, 50–100 nL) was microinjected in the medullary raphe nuclei in order to map its effects on swallowing (Figure 5). This structure was chosen because it contains serotonergic neurons as well as non-serotonergic neurons, harboring 5HT2A receptors and innervating numerous medullary nuclei, including the NTS [28]. Overall, 118 injections were made at 18 different sites in the caudal part of raphe nuclei (*n* = 15). The mean number of tracks was 3 ± 1, and the mean number of injections per track was also 3 ± 1 per preparation. These microinjections produced either no change (*n* = 86) or an increase (*n* = 32) in SR compared to control conditions (Table 2).

Similarly to systemic QPZ experiments, all swallows observed after QPZ microinjections in raphe nuclei were isolated (Figure 5A). To evaluate the effects of these microinjections on SR (and the latency to the first swallow), we used two different methods. First, regardless of the injection site, all successful injections leading to an increase in SR in a single preparation (range 1–4) were used to measure the mean SR per rat. Averaged QPZ group values revealed a significant increase in SR compared to control values (0.8 ± 0.7 vs. 2.2 ± 0.5, *p* < 0.001, in control vs. QPZ conditions, respectively; Figure 5C; Table S10). SR was significantly increased for the first minute post-injection, but not over the 5 min post-injection period, compared to the control. The mean latency to the first swallow was 14 ± 11 seconds (Table S10). Then, we further analyzed the effects of QPZ on swallowing per site of injection. To do so, we counted the total number of injections made at a given site in our different rats and determined the number of successful (and unsuccessful) injections (Table 2).

A map representing the 18 sites of injections and the different effects of QPZ observed at each site is illustrated in Figure 5B. In most sites (12/18), QPZ injections did not change SR. However, successful injections were observed at six adjacent sites within the caudal aspect of the raphe nuclei encompassing the raphe pallidus and raphe obscurus. Consistent data were particularly obtained at three sites, where more than eight injections were made and 50% of them (or more) resulted in an increase in SR.

Table 3 reports the mean SR measured after QPZ injection at these sites. With this method of evaluation, SR at each of the three sites varied from 2.3 to 3.0 swallows per min, and group values showed a significant increase in SR at one minute, but not over the entire 5 min post-injection period (Table 3). Of note, the systemic or central route of QPZ injection resulted in similar SR (2.3 ± 0.8 vs. 2.7 ± 0.4 sw/min, respectively, *p* = 0.19), although the systemic route produced longer effects than the central route. The latency of the first swallow found with this method was 9.1 ± 3.9 sec. Interestingly, QPZ microinjections in the medullary raphe nuclei had no effect on Rf (30 ± 10 vs 30 ± 8 cycles/min, in control vs. QPZ groups, respectively; *p* = 0.34) or on BP (78 ± 23 mmHg for both groups).

## 3. Discussion

This study demonstrated that the serotonergic agonist QPZ enhanced swallowing in situ. Furthermore, injection of the 5-HT2 receptor antagonist methysergide prevented the stimulatory effect of QPZ on swallowing but was without effect on spontaneous swallows. Both spontaneous and QPZ-induced swallows were isolated and displayed similar motor patterns and relationships with central breathing. Contrary to the effects of NMDA, microinjection of QPZ in the NTS did not enhance swallowing. By contrast, microinjection of QPZ in the medullary raphe nuclei increased SR. Thus, we concluded that pharmacological stimulation of swallowing by QPZ in situ represents a useful model to further study the core circuitry of the swCPG, mechanisms of neuromodulation of swallowing, and swallow-breathing interactions.

### 3.1. Distribution of Serotonergic Receptors in Brainstem Respiratory and Swallowing Networks

Serotonin 5-hydroxytryptamine (5-HT) is a widespread monoaminergic neurotransmitter in the brain, and brainstem raphe nuclei represent the main source of 5-HT innervation to the brain and spinal cord. A large variety of 5-HT receptor (5-HTR) subtypes, ranging from 5-HTR1 to 5-HTR7, contribute to the neuromodulatory effects of 5-HT. Many structures belonging to the respiratory and swallowing networks receive dense 5-HT projections [29,30,31,32,33,34] and express a majority of 5-HTR1 and 5-HTR2, although other types of receptors have been described. For example, the NTS contains neurons expressing 5-HTR1a, 5-HTR1b, 5-HTR2a, 5-HTR2c [28,35,36], 5-HTR3 [37], 5-HTR4 [38] and 5-HTR7 receptor subtypes [39]. Axonal labeling has also been observed, notably in the solitary tract [40]. In the ventral medulla, neurons of the pre-Bötzinger complex express 5-HTR1a, 5-HTR2a and 5-HTR4a receptors [41,42,43]. In the dorsolateral pons, parabrachial neurons express 5-HTR1a, 5-HTR2a, 5-HTR2c and 5-HTR3 receptors [44]. In addition, cranial and spinal motor nuclei involved in breathing and/or swallowing harbor dense 5-HT axon terminals. Hypoglossal motoneurons express 5-HTR1a, 5-HTR1b and 5-HTR2a receptors [45]. These receptors are also retrieved in the nucleus ambiguus, where laryngeal and pharyngeal motoneurons are located [45,46,47]. Phrenic motoneurons are known to express multiple serotonergic receptors, including 5-HTR1b, 5-HTR2a and 5-HTR2c [48]. Therefore, such a large number of structures expressing a diversity of 5-HTR subtypes likely mediate multiple functional effects induced by 5-HT or serotonergic mimetics such as QPZ.

### 3.2. Specificity and Functional Effects of Quipazine

Quipazine is a predominantly 5-HTR2a agonist [49,50,51], and it may have a potential agonist activity at 5-HTR1b [52,53,54]. Quipazine also acts as a potent antagonist at peripheral 5-HT3 receptors [49,55,56]. Interestingly, 5-HT2a receptor stimulation by QPZ has been used to induce motor activity and promote recovery of function after spinal cord injury in rodents [57,58]. Quipazine also promotes fictive locomotion via stimulation of 5-HT2a receptors on in vitro spinal cord preparations from newborn rats [59]. At a cellular level, QPZ excites both interneurons and motoneurons of the spinal locomotor networks through 5HTR2a stimulation [60,61]. Of note, reports in rodents suggested that QPZ has no affinity for 5HTR1a autoreceptors which are classically coupled to inhibitory G proteins [51,59,61]. Thus, it is reasonable to assume that elicitation of fictive swallowing after systemic QPZ injection in situ was mainly achieved via 5-HTR2a stimulation at the central level. The blockade of this stimulatory effect by the 5-HTR2 antagonist methysergide is in agreement with this assumption.

Numerous experiments demonstrated an important role for the 5HTR2a in central respiratory control [62,63,64,65,66] and cardiovascular regulation [47,67,68], in accordance with a moderate to strong somatodendritic 5-HTR2a expression in medullary structures involved in cardiorespiratory control, including cranial motoneurons [69]. Activation of 5-HTR2a induced a membrane depolarization and increased firing frequency of both phrenic and hypoglossal motoneurons [70,71,72], expiratory interneurons of the ventral respiratory column [73], as well as sympathetic preganglionic neurons [74]. Interestingly, intravenous injection of a serotonergic agonist acting at 5-HTR2a (and 2c) improved upper airway stability and also elevated blood pressure (BP) [75]. Thus, the increases in Rf and BP observed herein after systemic QPZ injection may result from a broad stimulation of 5HTR2a expressed by medullary neurons of the cardiorespiratory networks. Our results showed no significant correlation between changes in Rf or BP and SR after systemic QPZ injection. Thus, we believe that enhancement of swallowing after systemic QPZ was not a secondary side effect originating from alteration of cardiorespiratory functions. Distinct medullary structures may mediate the different functional effects of QPZ. This view is reinforced by our results showing that microinjection of QPZ in medullary raphe nuclei also increased SR but had no effect on Rf and BP.

### 3.3. Effects of QPZ on Swallowing

Previous studies on serotonergic modulation of swallowing reported conflicting results in anesthetized rats. Stimulatory effects of various serotonergic agonists such as 5-HT, CPP and QPZ were first reported [5], whereas Kessler and Jean [21,22] showed inhibitory effects of monoamines including noradrenaline, 5HT and QPZ and suggested the existence within the NTS of a serotonergic inhibition of the swallowing reflex elicited by laryngeal afferents. Discrepancies may be due to the use of different doses of serotonergic agonists, but also different anesthetics between these studies [19]. The excitatory effect of QPZ on swallowing found in our experimental model without anesthesia confirmed earlier observations [5]. However, a similar dose of QPZ (1–2 µM/kg) produced a shorter effect in situ (15 min, present results) than in vivo (up to hours) [5]. Similarly, a faster SR was reported in vivo (up to 7–8 swallows per min). A lower temperature and/or a decrease in drug bioavailability may be responsible for this reduced effect of QPZ in situ.

All of the spontaneous and QPZ-induced swallows were isolated, and showed similar types of swallow and motor patterns, with a similar delay between the starts of the XII and X swallowing bursts. This physiological sequence of motor events characterizes the pharyngeal phase of swallowing [7,8]. Interestingly, the 5-HTR2 antagonist methysergide efficiently blocked the stimulatory effect of QPZ on swallowing but did not suppress all swallows. These results suggest that QPZ may increase excitability of the swCPG via 5-HTR2 stimulation. They also suggest that the blockade of 5-HTR2 has no effect on mechanisms involved in spontaneous (or reflexive) swallow production. Spontaneous swallows in situ have been interpreted as being due to an incomplete descending inhibitory gating from the pons [12,16]. We do not exclude, however, the possibility that fluid penetration (or small movements of fluid) in upper airways produced a tonic excitation of swallowing-related peripheral afferents, and resulted in low numbers of reflexive (instead of spontaneous) swallows in our preparations.

### 3.4. Swallow-Breathing Coordination In Situ

Single but not repetitive swallows were produced after QPZ injection, allowing swallow-breathing coordination and the effects of swallowing on central respiratory activity to be analyzed in situ. This method of pharmacological elicitation of isolated swallows represents an advantage over sustained electrical stimulation of the superior laryngeal nerves or injection of water into the pharyngeal cavity which resulted in repetitive swallows [8,10,12,13]. Most swallows were produced near the phase transition between central inspiration and the post-inspiratory phase of breathing, in agreement with previous experimental data in vivo [8,9] and in situ [12,16]. However, there was no significant change in duration of central inspiration or expiration by Post-I or Exp swallows, respectively. This is in contrast with recent results obtained in ventilated animals [8], but in agreement with a previous observation during stage 2 auto-resuscitation in situ [16]. Fictive breathing in situ is not associated with rib cage movement, airflow, vagal feedback from the lungs, muscle feedback or changes in glottal or sub-glottal pressure. The present data suggest a contribution of these parameters in swallow-breathing interactions. This is in line with evidence showing a negative modulation of swallow initiation by vagal feedback and a resetting by lung inflation (peripheral gating), highlighting the important role of vagal pulmonary afferent activity in swallow-breathing coordination [8]. Future in situ experiments using artificial ventilation applied through the upper airway or a tracheal tube may help to further explore the role of these parameters.

### 3.5. Is the NTS Involved in Serotonergic Modulation of Swallowing?

The NTS is supposed to contain the second-order sensory neurons and the core circuitry forming the swallowing central pattern generator (swCPG) responsible for the purely reflexive pharyngeal phase of swallowing [2,76,77,78]. Deglutition can be triggered by activating either NMDA or non-NMDA receptors localized within the NTS, suggesting that both receptor subtypes are involved in swallowing elicited under physiological conditions [18]. Consistent with this observation, our results showed that repetitive swallows with central apnea were readily evoked after microinjection of NMDA within the swallow trigger zone in the NTS. However, no swallow could be induced after microinjection of QPZ in the active NTS sites, despite the expression of 5-HTR (including 5-HTR2a) in NTS neurons [28,40]. Therefore, our results suggest that 5-HTR2a stimulation of NTS neurons is unable to initiate and/or enhance swallowing. This markedly contrasts with the conclusions from findings in anesthetized rats, although 5-HT but not QPZ was microinjected in the NTS [19]. Further experiments are required to clarify this apparent complex serotoninergic neuromodulation of swallowing at the level of the NTS.

### 3.6. Medullary Raphe Nuclei May Modulate Swallowing

Previous work by Jean and Kessler [22] showed that electrical stimulation of several brainstem structures overlapping serotonergic regions, such as the nucleus raphe magnus and the nucleus raphe pallidus, induced an inhibition of swallowing. Opposite results were obtained in our study, since microinjection of QPZ in the caudal aspect of medullary raphe nuclei, including the raphe obscurus and pallidus, increased SR. Methodological differences between the two studies may explain these conflicting results. Brainstem raphe nuclei contain distinct groups of neurons, i.e., serotonergic and non-serotonergic cells, harboring different serotonergic receptors, and are supposed to play distinct functional roles (see below) [28]. Given this heterogeneity of neurons, electrical stimulation of the raphe nuclei may not represent an appropriate method to study serotonergic modulation of swallowing. On the contrary, it is conceivable that drug microinjection in raphe nuclei exerted a more selective stimulation of neuronal sub-types, unraveling a serotonergic stimulatory effect on swallowing. Raphe nuclei have been suggested to control the cough reflex [79]. To our knowledge, the present study is the first to suggest a direct implication of medullary raphe nuclei in the control of swallowing.

Serotonergic neurons of the medullary raphe nuclei mostly express 5-HT1a somatodendritic autoreceptors responsible for an inhibition of serotonergic transmission, whereas non-serotonergic raphe neurons harboring the excitatory 5-HTR2a have been involved in autonomic functions including the control of breathing [28,80]. Interestingly, serotonergic and non-serotonergic medullary raphe neurons displayed distinct electrophysiological properties [81,82], and many non-serotonergic (mesencephalic) raphe projection neurons are glutamatergic [83]. Therefore, distinct afferent inputs to the NTS from the raphe nuclei could exert a differential neuromodulation within the respiratory and swallowing networks. We suggest that QPZ may stimulate post-synaptic 5-HTR2a of serotonergic or more likely non-serotonergic raphe neurons, which in turn may excite the NTS neurons forming the swCPG. Further investigations are needed to verify this hypothesis, notably to demonstrate an increased excitability of raphe neurons during swallowing, precise their phenotype and identify their afferent and efferent projections.

### 3.7. Functional Relevance of Findings

Raphe nuclei have a major role in the control of cranial and spinal motoneuron excitability and autonomic function, including breathing [24]. This study suggests a role for the caudal medullary raphe nuclei in serotoninergic control of swallowing, although precise conditions that could stimulate raphe nuclei to increase swallowing remain to be identified. Our results also reinforce the view that raphe nuclei represent an important structure extrinsic to the swCPG that could operate as a link between higher CNS structures and the brainstem swallowing network [76]. For example, if the raphe regions identified herein receive projections from swallowing-related cortical areas and send efferent projections to the swCPG in NTS, they might be involved in volitional control of swallowing. Loss of medullary serotonergic raphe neurons occurs in multiple system atrophy [24], and patients with this disease also suffer from dysphagia [25]. In addition, breathing and feeding difficulties are frequently observed in Prader–Willi syndrome (PWS) and Shaaf–Yang syndrome [84], and several alterations in raphe nuclei have been demonstrated in mice models for PWS [85,86,87]. Better knowledge of serotonergic modulation of swallowing, such as identification of new targets, mechanisms of action, and serotonergic receptor subtypes, would be particularly useful to find new therapeutic treatments for patients suffering from dysphagia.

## 4. Materials and Methods

Rats were handled and cared for in accordance with the Guide for the Care and Use of Laboratory Animals (N.R.C., 1996), the European Communities Council Directive of September 22th 2010 (2010/63/EU, 74), as well as the French laws. Since all experiments were performed post-mortem, experimental protocols did not need accreditation from the French Ministry of Agriculture.

### 4.1. Working Heart-Brainstem or In Situ Preparation

The experiments were performed in the arterially perfused working heart-brainstem (in situ) preparation as described originally by Paton [88]. Juvenile *Wistar* rats (P15-P21; *n* = 42; body weight 50–110 g; Janvier Labs, Le Genest St Isle, France) were deeply anesthetized with isoflurane (1-chloro-2,2,2-trifluoroethyl-difluoromethylether; Baxter). The level of anesthesia was assessed by a lack of response to noxious pinches of the hindpaw or the tail. Once both cardiac and respiratory activities were depressed, animals were transected below the diaphragm and decerebrated at the pre-collicular level. The preparation was then immersed in cool (5 °C) artificial cerebrospinal fluid (aCSF) gassed with carbogen (95% O_2_ and 5% CO_2_; pH 7.35) and skinned. The composition of aCSF was as described previously [7]. Lungs, but not the heart, were systematically removed, the descending aorta was isolated, and the right phrenic, vagal and hypoglossal nerves were dissected free from surrounding tissues and prepared for recording. The preparation was moved to a recording chamber, where the descending aorta was cannulated and perfused with warm (32 °C) oxygenated aCSF using a peristaltic pump (Watson-Marlow, Falmouth, Cornwall, UK; model 520s). The perfusate was filtered and continuously recirculated. Flowrates (20–28 mL/min) were adjusted to obtain a stable eupneic pattern of breathing, as evidenced by a ramp-like inspiratory activity recorded from the phrenic nerve, a sharp transition between inspiration and expiration, and by a post-inspiratory discharge activity recorded from the vagal nerve. The perfusion pressure (50–125 mmHg) was measured via a double lumen cannula connected to the descending aorta and a blood pressure monitor (World Precision Instrument, Sarasota, Fl, USA).

### 4.2. Recording and Analyses of Cardiorespiratory and Swallowing Parameters

Motor activities were recorded from the phrenic (Phr), vagal (X) and hypoglossal (XII) nerves using suction electrodes mounted on micromanipulators. Raw and integrated (time constant 100 ms) signals were amplified and filtered (gain 500–10000; BP 300–3000Hz) before being digitized (12 KHz, 16 bits). Signals corresponding to blood pressure (BP) and electrocardiogram (ECG) were also recorded and digitized (Tucker-Davis Technologies, Alachua, Fl, USA). Digitized signals were visualized and stored on a PC for later processing (OpenEx softwares, Plexon Inc., Dallas, TX, USA). In all experiments, stable respiratory rhythm with a clear three-phase pattern on nerve activities was detected 20 to 30 min after onset of reperfusion. This pattern included a pre-inspiratory XII activity, a ramp-like Phr activity and a typical decrementing vagal discharge indicative of the post-inspiratory phase of breathing. Swallowing consisted in short-lasting (300–500 ms) bursts on the XII and X nerves, corresponding to normal outflow to tongue and laryngeal muscles, respectively [7,8,12].

Several criteria were used to precisely identify and quantify respiratory and swallowing parameters from integrated nerve signals, as partially described in previous studies [7,8,89]. First, swallows were characterized by concomitant bursts on cranial nerves, with a slight precession of the XII burst which corresponded to the normal sequence of motor events. Second, swallow-related bursts not associated with respiratory activity on Phr, XII and X nerves were labeled as to expiratory-type swallows (Exp Sw). Those immediately preceded by early expiration (or post-inspiration) corresponded to post-inspiratory type swallows (Post-I Sw). The start of the Post-I Sw was always marked by a clear indentation (almost reaching the baseline) on the integrated XII signal. An indentation was also seen on the integrated X trace toward the beginning of Post-I swallow. This indentation was always preceded by a peak corresponding to respiratory (i.e., post-inspiratory) discharge, and followed by another peak corresponding to swallow-related discharge.

A semi-automatic thresholding method was applied to each integrated trace to detect and mark the rising edge and/or peak of each respiratory or swallowing burst (Offline Sorter, Plexon Inc., Dallas, TX, USA Ver2). The peak markers were then used as triggers to compute averaged envelopes, and measure burst and/or cycle duration (in seconds), as well as peak amplitude and area under the curve (AUC) in arbitrary units (NeuroExplorer, Plexon Inc., Dallas, TX, USA Ver3.266). For respiratory parameters, inspiratory and/or post-inspiratory nerve activities were measured before and after drug application using 20 to 50 respiratory cycles. These cycles were made of consecutive breaths in control condition, but not after drug application due to more frequent swallow-breathing cycles. For swallow parameters, the total duration of both Post-I and Exp swallows were defined as the delay between the start of the integrated XII discharge and the end of the integrated X discharge. Whatever the type of swallow, the start and the end of the XII burst were constantly used to measure the duration and AUC of the XII burst (Figure S1). A similar method was used to compute the duration and AUC of Exp swallows. Due to the overlap between respiratory and swallowing discharges of the X nerve, we developed an original method to provide accurate measurements of the duration and AUC of the X burst during Post-I swallows (Figure S1A). Indeed, we noticed that if the indentation clearly visible on the X envelope toward the beginning of swallowing was used to mark the start of the X swallow burst, it resulted in reduced duration and AUC of Post-I X bursts compared to values measured during Exp swallows (Figure S1B). This was considered an artefact, because the duration (and AUC) of the XII bursts and the total duration of swallow were almost similar between the two types of swallows. Thus, we used a graphical method to estimate the start of the Post-I X burst (Figure S1C). The rising edge of the Post-I X burst was used to draw a tangent line from the peak, through the indentation, down to the baseline of the integrated trace. The crossing point between the tangent line and the baseline was used to define the (estimated) start of the X burst. The resulting construct formed a triangular envelope delineating the Post-I swallow-related X burst, from which consistent measurements of burst duration and AUC were made.

### 4.3. Pharmacological Induction of Swallowing

A few spontaneous swallows have been observed in situ when preparations displayed a eupneic pattern of breathing [16]. To enhance the number of swallows in situ, we used a pharmacological method previously described in the rat in vivo [5], consisting in systemic injection of the serotonergic agonist quipazine (QPZ, quipazine maleate, Sigma, Saint-Quentin Fallavier, France). The drug concentration was first assessed in preliminary experiments by varying the dose of QPZ dissolved in the aCSF (range 0.5–10 µM/Kg). Swallowing was enhanced with 1 and 2 µM/Kg drug concentrations, and higher doses did not produce stronger or longer effects. Therefore, we choose a concentration of 1.5 µM/Kg of QPZ in the present experiments, because it not only corresponded to the optimal drug concentration found in our preliminary study, but also matched the doses used in vivo [5]. In order to evaluate the low production of swallow in control conditions, i.e., spontaneous events without drug, the number of swallows was counted in each rat over 10 or 15 min, and results were expressed as swallows per min and averaged per 5 min intervals. After this control period, QPZ was added to the aCSF (systemic injection), and the number of swallows was counted over 15 to 20 min, and results were also expressed as mean SR per 5 min intervals and per rat. Other experiments (*n* = 5) were performed to test the ability of the 5-HT2-receptor antagonist methysergide (methysergide bimaleate, Sigma; 1 µM/kg in aCSF) to block QPZ-induced swallowing. In those experiments, after an initial 10–15 min control period, QPZ was injected in aCSF and SR was determined for 5 min. Then, methysergide was added to the perfusate and SR was recorded for 15 to 20 min after drug injection. Other experiments (*n* = 3) were performed to test the effect of methysergide alone on swallowing. Swallow rate was measured for 15 min before and after injection of methysergide (1 µM/kg). Two series of experiments were designed to study the central effects of QPZ on swallowing. In the first series, we performed microinjections of QPZ (3 mM, 50–200 nL) into the NTS (*n* = 5) using a nanoinjector (WPI, USA) mounted on a micromanipulator (nanostepper), and a microcontroller allowing flowrate and volume adjustments (5–10 nL/s, WPI, USA). The proper placement of the pipette into the NTS swallowing “trigger zone” was first assessed by microinjection of N-methyl-D-aspartate (NMDA, 1 mM, 10–50 nL; *n* = 5), as previously reported [18]. Because NMDA but not QPZ induced swallowing, we did not quantify SR in this series. In the second series, we mapped the effects of QPZ microinjections (3 mM, 50–100 nL) within the caudal medullary raphe nuclei (*n* = 15). In these experiments, the control SR was measured for 15 min before the injection protocol. The nanoinjector was positioned perpendicular to the floor of the fourth ventricle and moved along the midline from 1 to 3 mm rostral to the obex with 0.5 mm steps. Then the pipette was inserted through the dorso-ventral axis of the medulla at depths between 1.25 to 2.25–2.75 mm below the surface with 0.5 mm steps. In each rat, several tracks were performed at different positions along the midline. A single track was made at a given midline position, and 3 to 4 injections were made per track. Only one injection was made at a given depth, and a 5 min period was always respected between 2 injections. This delay was chosen because we noticed a rather short-lasting effect during the experiments, which was confirmed by offline analyses. For each site of injection, results are reported as a success or a failure to increase SR for the first minute post-injection (although SR was also measured over the 5 min period post-injection). Thus, each success or failure was attributed based on a single microinjection. For successful injections, the latency between the end of injection and the occurrence of the first swallow was also determined. All successful injections observed in an individual were used to calculate the mean SR per rat, and the mean latency to the first swallow. To further evaluate the potency of QPZ to increase swallowing at a given site, the total number of injections made at the same site in different rats was calculated, and numbers of successes and/or failures were determined. Each site was represented as a pie chart with a different color to depict the effects on swallowing, and a different size according to the total number of injections. For those sites where both successes and failures were observed, the colored areas within pie charts represented the percentages of successes and failures.

### 4.4. Experimental Protocol and Parameter Analyses

In each experiment, parameters related to spontaneous fictive breathing, BP, heart rate (ECG), and swallowing started to be recorded ~45 min after onset of reperfusion. Control cardiorespiratory parameters and the rate of spontaneous swallows were measured over an initial 15 min recording period. Then, the parameters were recorded over a longer period (~30–45 min) to analyze the effects of systemic or central QPZ injections, or the effects of methysergide, on cardiorespiratory and swallowing functions. Integrated activities of the Phr, X and XII nerves were marked and coded for measurements of breathing and swallow motor output (total duration of burst activity, peak amplitude, area under the curve, the delay between the starts and peaks of the XII and X bursts, as previously described) [8,11,89]. Overall, analyses were done using spontaneous (*n* = 233) and QPZ-induced swallows (*n* = 307) obtained in the same preparations (*n* = 16). The swallow duration was defined as the interval between the start of the swallowing-related XII nerve burst activity and the end of the swallowing-related X nerve burst activity. For analysis of swallow-breathing coordination in control and QPZ conditions, the duration of the normal respiratory cycle was first calculated from at least 15 successive respiratory cycles without swallowing. As both spontaneous and QPZ-induced swallows always resulted in isolated and not repetitive swallows (see Results), the occurrence of each isolated swallow was marked within each breathing cycle containing a swallow (i.e., swallow-breathing cycle). Analyses of the phase-relationship between breathing and swallowing were performed as described recently [8]. Durations of both inspiratory and expiratory phases of the swallow-breathing cycle were measured and normalized to control respiratory cycle values. Therefore, the occurrence of each swallow was expressed as a percentage of the control-normalized duration of the inspiratory or expiratory phase of breathing. Swallows that appear within 0%–20% of the normalized expiratory duration were labelled Post-I swallows, whereas the others occurring later during the expiratory phase (i.e., within 21%–100% of the normalized expiratory duration) were labelled Exp-swallows, as previously described [8].

### 4.5. Statistical Analyses

Results were averaged per preparation and per group. Amplitudes and areas under the curve (AUC) of integrated nerve activities, as well as duration of inspiration and expiration of the swallow-respiratory cycles, were normalized. Normality of the values was tested using the Shapiro–Wilk test. Variables with normal distribution were analyzed using paired *t*-tests or an analysis of variance (ANOVA), when two or three groups were compared, respectively. When normality was not verified, continuous variables were compared using the nonparametric paired Wilcoxon test or the Kruskal–Wallis test (followed by Dunn’s post-hoc tests). A Pearson’s Chi-squared test with Yates’ continuity correction with a 2 × 2 contingency table was used to compare the distribution of swallows within the swallow-breathing cycle between control (spontaneous swallows) and QPZ groups. Comparisons of SR obtained in control, QPZ and methysergide groups were performed with ANOVA followed by Dunnett’s post-hoc tests. Pearson’s correlation tests were used to compare the magnitude of change in SR with change in either Rf or BP elicited by QPZ. Results are expressed as mean ± standard deviation (SD). Values were considered significant when *p* < 0.05.

## Figures and Tables

**Table 1:** Quantification of swallowing-related bursts.

	Hypoglossal Nerve		Vagus Nerve		
	Control	QPZ	Control	QPZ	
Normalized Amplitude (%)	100	101 ± 19	100	97 ± 9	ns
Normalized area under the curve (%)	100	107 ± 19	100	98 ± 13	ns
Duration (s)	0.56 ± 0.12	0.51 ± 0.14	0.43 ± 0.08	0.43 ± 0.06	ns

Data are expressed as mean ± SD, ns: not significant, Wilcoxon matched-pairs signed rank test or paired *t*-test for nonparametric or parametric data, respectively.

**Table 2:** Number of injections per site and effects of quipazine on swallowing after drug microinjection in the medullary raphe nuclei.

	Obex (mm)				
Depth (mm)	+1	+1.5	+2	+2.5	+3
1.25	(6)	**3** (11)	(11)	(1)	(2)
1.75	(7)	**9** (5)	**5** (9)	(1)	(2)
2.25	(7)	**8** (6)	**6** (6)	(1)	(1)
2.75	---	---	**1** (8)	(1)	(1)

Numbers indicated in parenthesis or in bold represent injections associated with no effect or an increase in swallow rate, respectively.

**Table 3:** Changes in swallow rate (SR) after microinjections of quipazine (QPZ) at the most successful sites in raphe nuclei. SR was measured for 5 min in control, and for 1 and 5 min after QPZ injection.

Successful Sites (mm)		Swallow Rate (sw/min)			
From Obex	Depth	Control	QPZ 0–1 min	QPZ 0–5 min	Latency
+1.5	−1.75	0.8	2.9	2.0	7.8
+1.5	−2.25	0.8	3.0	2.1	6.0
+2	−2.25	1.4	2.3	2.0	13.4
	Mean ± SD	1.0 ± 0.3	2.7 ± 0.4	2.2 ± 0.1	9.1 ± 3.9

## Supplementary Materials

Supplementary materials can be found at https://www.mdpi.com/1422-0067/21/14/5120/s1. Figure S1. Schematic illustration of methods used to identify and quantify swallow-related bursts in situ. Table S1. Respiratory frequency (Rf) before and after systemic quipazine (QPZ) injection at 15 min, and latency to the first swallow after drug injection. Data are expressed per 5 min or 15 min intervals. Table S2. Of the inspiratory (Ti) and expiratory (Te) phases of breathing before and after systemic quipazine (QPZ) injection. Table S3. Blood pressure (BP, mmHg) before and after systemic quipazine (QPZ) injection, and percentage of increase (% Inc) between conditions. Table S4. Hear rate before and after quipazine (QPZ) injection. Table S5. Detailed comparisons of swallowing-related bursts before and after quipazine injection (QPZ). Duration of swallows, swallowing-related bursts and the delay between the starts of the hypoglossal and vagal nerves (Delay) are expressed in seconds, whereas burst amplitude and the area under the curve (AUC) are expressed in percentage of Control values. Table S6. Swallow rate (SR) before and after systemic quipazine (QPZ) injection at 15 min, and latency to the first swallow after drug injection. Data are expressed per 5 min or 15 min intervals in control and QPZ conditions. Table S7. Magnitude of increase in BP and Rf before and after systemic quipazine (QPZ) injection, and ratio between the mean swallow rate (SR) after QPZ injection and the mean SR in control condition (means ratio). Table S8. Swallow rate in control condition, after injection of quipazine alone (QPZ), and after methysergide (Methy) injection in the same preparation. Data are expressed per 5 or 15 min intervals. Table S9. Swallow rate and respiratory frequency before and after methysergide injection. Parameters were measured for 15 min before and after systemic drug injection. Table S10. Swallow rate (SR) before and after quipazine (QPZ) microinjection in raphe nuclei, and latency to the first swallow after drug injection. SR was measured for 5 min in control, and for 1 and 5 min after QPZ injection using successful trials eliciting swallows after drug injection.
