# Supplementary material for: Quipazine Elicits Swallowing in the Arterially Perfused Rat Preparation: A Role for Medullary Raphe Nuclei?
Source: Int J Mol Sci. 2020 Jul 20;21(14):5120. doi: 10.3390/ijms21145120 (PMC7404031; doi:10.3390/ijms21145120)
Supplement: Supplementary file 1 [file ijms-21-05120-s001.pdf]

International Journal of *Molecular Sciences*

MDPI

Quipazine Elicits Swallowing in the Arterially Perfused Rat Preparation: A Role for Medullary Raphe Nuclei?

Victor Bergé-Laval [1]. Christian Gestreau [1]

Aix-Marseille Université and INSERM, Institut de Neurosciences des Systèmes, UMR 1106, Bd Jean Moulin, 13005 Marseille, France

Supplementary Materials

Table S1. Respiratory frequency (Rf) before and after systemic quipazine (QPZ) injection at 15 min, and latency to the first swallow after drug injection. Data are expressed per 5 min or 15 min intervals.

	Rf (cycles/min)									
	Control			QPZ				Mean Control	Mean QPZ	
	Time (min)							Time (min)		Latency
**Preparation #**	**0–5**	**5–10**	**10–15**	**15–20**	**20–25**	**25–30**	**30–35**	**0–15**	**15–30**	**(s)**
**1**	---	22	19	14	18	22	---	21	18	38
**2**	16	16	14	22	30	29	25	15	27	96
**3**	20	20	21	21	20	21	20	20	21	---
**4**	11	11	12	19	25	23	---	11	22	149
**5**	27	28	34	14	4	23	31	30	14	139
**6**	---	19	20	16	16	21	18	20	18	---
**7**	---	49	47	71	69	45	---	48	62	128
**8**	---	29	31	35	35	32	30	30	34	---
**9**	---	24	29	39	52	51	---	27	47	76
**10**	21	22	24	29	32	30	28	22	30	133
**11**	---	17	16	33	39	30	---	17	34	152
**12**	18	18	17	19	20	20	15	18	20	---
**13**	36	36	36	55	65	61	---	36	60	96
**14**	23	25	23	69	32	27	---	24	43	65
**15**	15	20	14	16	15	13	13	16	15	127
**16**	---	17	17	15	14	14	14	17	14	103
**Mean ± SD**	21 ± 7	23 ± 9	23 ± 10	30 ± 19	30 ± 18	29 ± 13	22 ± 7	23 ± 9	**30 ± 17 ***	108 ± 36

* *$p < 0.05$, Wilcoxon matched-pairs signed rank test.*

Table S2. of the inspiratory (Ti) and expiratory (Te) phases of breathing before and after systemic quipazine (QPZ) injection.

	Duration (s)			
	**Control (0–15 min)**		**QPZ (0–15 min)**	
Preparation #	Ti	Te	Ti	Te
1	0.91	1.93	1.22	2.58
2	1.16	2.56	1.05	1.31
3	0.82	2.09	0.93	2.03
4	1.42	3.99	1.16	1.97
5	0.57	1.32	0.83	1.31
6	0.70	2.26	0.72	2.77
7	0.25	0.46	0.40	0.76

8	0.56	1.32	0.50	1.11
9	1.02	1.08	0.60	0.55
10	0.48	1.76	0.43	1.44
11	1.29	2.59	0.93	1.26
12	0.59	2.73	0.56	2.56
13	0.90	0.59	0.72	0.18
14	0.58	1.81	0.54	1.12
15	0.56	2.32	0.92	3.44
16	0.71	2.84	0.69	3.43
Mean ± SD	0.78 ± 0.32	1.98 ± 0.90	0.76 ± 0.25	1.74 ± 0.99

Table S3. Blood pressure (BP, mmHg) before and after systemic quipazine (QPZ) injection, and percentage of increase (% Inc) between conditions.

**Preparation #**	**BP Control**	**BP QPZ**	**% Inc**
1	116	160	38
2	49	65	33
3	83	97	17
4	53	71	34
5	197	285	45
6	75	95	27
8	54	73	35
9	45	48	7
10	108	120	11
11	75	95	27
12	51	62	22
13	60	75	25
14	120	149	24
16	125	131	5
Mean ± SD	87 ± 43	109 ± 61 ***	25 ± 12

*** *$p < 0.001$, Wilcoxon matched-pairs signed rank test.*

Table S4. Hear rate before and after quipazine (QPZ) injection.

	**Heart Rate (bpm)**	
**Preparation #**	**Control**	**QPZ**
**1**	347	353
**2**	348	339
**3**	284	309
**4**	424	411
**5**	320	295
**Mean ± SD**	345 ± 51	341 ± 45

$p = 0.73$, Paired t-test.

Table S5. Detailed comparisons of swallowing-related bursts before and after quipazine injection (QPZ). Duration of swallows, swallowing-related bursts and the delay between the starts of the hypoglossal and vagal nerves (Delay) are expressed in seconds, whereas burst amplitude and the area under the curve (AUC) are expressed in percentage of Control values.

	**Duration (s)**			
	Vagus Nerve		Hypoglossal Nerve	
Preparation #	Control	QPZ	Control	QPZ

1	0.32	0.40	0.49	0.45
2	0.38	0.41	0.64	0.47
3	0.43	0.41	0.62	0.45
4	0.39	0.34	0.60	0.40
5	0.43	0.53	0.49	0.61
6	0.57	0.37	0.47	0.39
7	0.47	0.45	0.52	0.51
8	0.36	0.43	0.33	0.35
9	0.56	0.43	0.74	0.68
10	0.42	0.52	0.67	0.77
Mean ± SD	0.43 ± 0.08	0.43 ± 0.06	0.56 ± 0.12	0.51 ± 0.14

Stat : cf Table 1.

Preparation #	**AUC (% from Control)**		**Amplitude (% from Control)**	
	Vagus Nerve	Hypoglossal Nerve	Vagus Nerve	Hypoglossal Nerve
1	85	105	99	85
2	100	100	100	98
3	102	100	101	102
4	99	143	108	121
5	100	100	86	72
6	100	100	98	100
7	112	135	98	139
8	66	79	108	90
9	100	100	80	103
10	112	112	95	101
Mean ± SD	98 ± 13	107 ± 19	97 ± 9	101 ± 19

Stat : cf table 1.

Preparation #	**Swallow Duration (s)**		**Delay (s)**	
	Control	QPZ	Control	QPZ
1	0.55	0.53	0.045	0.046
2	0.40	0.39	0.041	0.029
3	0.49	0.61	0.026	0.027
4	0.46	0.46	0.059	0.055
5	0.47	0.39	0.061	0.058
6	0.52	0.51	0.042	0.044
7	0.74	0.68	0.046	0.048
8	0.67	0.77	0.048	0.052
9	0.59	0.57	0.036	0.032
10	0.54	0.63	0.044	0.044
11	0.39	0.34	0.049	0.048
12	0.67	0.62	0.031	0.032
13	0.60	0.55	0.036	0.033
14	0.39	0.39	0.037	0.039
15	0.37	0.29	0.031	0.027
16	0.56	0.47	0.043	0.041
Mean ± SD	0.53 ± 0.11	0.52 ± 0.14	0.042 ± 0.01	0.041 ± 0.01

$p = 0.43$, Paired t-test *$p = 0.21$, Paired t-test*

Table S6. Swallow rate (SR) before and after systemic quipazine (QPZ) injection at 15 min, and latency to the first swallow after drug injection. Data are expressed per 5 min or 15 min intervals in control and QPZ conditions.

	**Swallow rate (swallows per min)**									
	Control			QPZ				Mean Control	Mean QPZ	
	Time (min)							Time (min)		Latency
Prepa-ration #	0–5	5–10	10–15	15–20	20–25	25–30	30–35	0–15	15–30	(s)
1	---	0.6	0.5	2.4	2	1.8	---	0.6	2.1	82
2	1	2.4	1.2	3	1	1.2	0.6	1.5	1.7	81
3	0.2	0.4	0.2	3	3.8	1.8	1	0.3	2.9	99
4	1.2	1.4	1.4	3	5.2	3.2	---	1.3	3.8	38
5	0.6	0.6	0.4	1	3	2.8	0.6	0.5	2.3	112
6	---	0.4	0.8	1.6	3.8	2.6	0.8	0.6	2.7	153
7	---	0	0.4	2.6	2.2	1.4	---	0.2	2.1	99
8	---	0	0.2	1.2	1.4	1.8	0.2	0.1	1.5	143
9	---	0	0.6	5.4	2.8	2.8	---	0.3	3.7	93
10	0.8	0.4	0.2	0.8	3.8	4	1.4	0.5	2.9	127
11	0.6	0.6	0.2	1.8	3.4	1.2	---	0.5	2.1	111
12	---	0.2	0	2.6	2	1	0.2	0.1	1.9	110
13	0.4	1	0.2	1	1.8	2	---	0.5	1.6	97
14	0.4	0.6	0.2	0.8	2.6	1.6	---	0.4	1.7	88
15	0	0.4	0.2	0.8	1.6	1.2	1.5	0.2	1.2	121
16	---	0.2	0	4.2	1.8	2.4	0.6	0.1	2.8	133
Mean ± SD	0.6 ± 0.4	0.6 ± 0.6	0.4 ± 0.4	2.2 ± 1.3	2.6 ± 1.1	2.1 ± 0.8	0.8 ± 0.5	0.5 ± 0.4	2.3 ± 0.8***	105 ± 28

**** $p < 0.001$, Dunn's multiple comparisons test.*

Table S7. Magnitude of increase in BP and Rf before and after systemic quipazine (QPZ) injection, and ratio between the mean swallow rate (SR) after QPZ injection and the mean SR in control condition (means ratio).

	**% Change from Control**		**Means Ratio**
Preparation #	BP	Rf	SR
1	38	-12	3.8
2	33	76	1.1
3	17	2	10.8
4	34	97	2.9
5	45	-54	4.3
6	27	-9	6.1
7	35	13	14.7
8	7	79	12.2
9	11	36	4.4
10	27	106	4.6
12	22	11	18.7
13	25	68	3.0
14	24	80	4.2
16	5	-14	28.0
Mean ± SD	25 ± 11.8	34 ± 49.8	8.5 ± 7.6

ns, Nonparametric Spearman correlation.

Table S8. Swallow rate in control condition, after injection of quipazine alone (QPZ), and after methysergide (Methy) injection in the same preparation. Data are expressed per 5 or 15 min intervals.

	**Control**	**QPZ**	**Methy**
Preparation #	0-15 min	0-5 min	0-15 min
17	0.57	2.4	0.6
18	1.53	3.0	0.3
19	0.27	3.0	1.2
20	1.33	3.0	1.9
21	0.67	1.9	0.6
Mean ± SD	0.87 ± 0.54	2.66 ± 0.50 *	0.92 ± 0.66

** $p < 0.05$, Dunnett's multiple comparisons test.*

Table S9. Swallow rate and respiratory frequency before and after methysergide injection. Parameters were measured for 15 min before and after systemic drug injection.

	**Swallow Rate**		**Respiratory Frequency**	
Preparation #	Control	Methysergide	Control	Methysergide
43	1.18	0.98	20	21
44	0.60	0.40	30	28
45	0.6	0.6	20	19
Mean ± SD	0.79 ± 0.34	0.66 ± 0.29	23 ± 6	23 ± 5

ns, Wilcoxon matched-pairs signed rank test.

Table S10. Swallow rate (SR) before and after quipazine (QPZ) microinjection in raphe nuclei, and latency to the first swallow after drug injection. SR was measured for 5 min in control, and for 1 and 5 min after QPZ injection using successful trials eliciting swallows after drug injection.

**Preparation #**	**Control**	**QPZ 0–1 min**	**QPZ 0–5 min**	**Latency (s)**
28	1.1	2.0	1.6	14
29	2.2	7.0	1.4	4
30	1.7	2.5	3.2	7
31	1.0	5.0	5.4	6
32	0.4	3.0	3.6	15
33	1.6	1.5	1.5	23
34	1.1	2.0	1.2	8
35	0.9	3.0	0.6	12
36	0.1	2.5	2.2	3
37	0.8	2.0	0.8	32
38	2.4	2.0	1.7	13
39	0.7	2.0	0.8	25
40	0.5	2.5	1.9	2
41	0.3	1.5	0.8	8
42	0.3	2.0	1.8	19
Mean ± SD	0.8 ± 0.7	2.2 ± 0.5***	1.3 ± 0.6	14 ± 11

** $p < 0.001$, Wilcoxon matched-pairs signed rank test.*

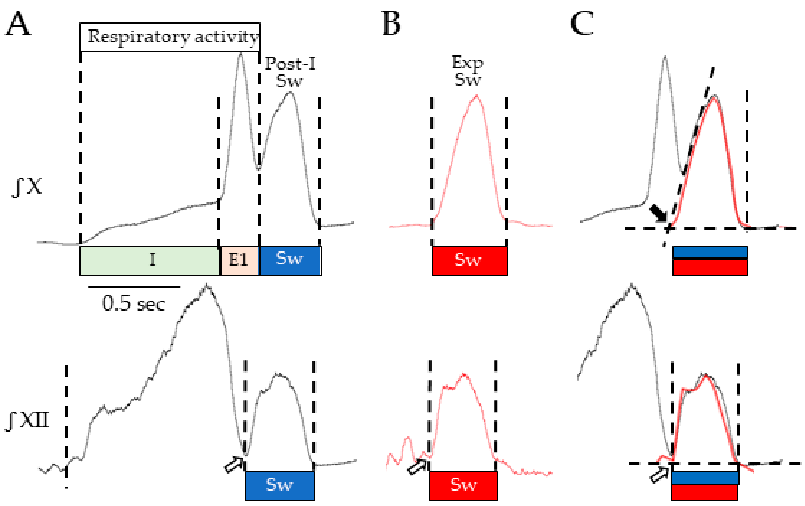

Figure S1. Schematic illustration of methods used to identify and quantify swallow-related bursts in situ. Averaged envelopes computed from integrated vagal (X) and hypoglossal (XII) nerve activities during post-inspiratory (Post-I Sw, n=10) (**A**), and expiratory swallows (Exp Sw, n=7) (**B**). Traces were obtained in control condition in the same preparation. Vertical dotted lines identify portions of the envelope corresponding to respiratory and/or swallowing activities. Inspiratory (I) and swallowing (Sw) XII bursts were easily differentiated. The start (open arrows) and the end (doted line) of the Sw XII bursts were clearly identified. Note the similar duration of the Sw XII burst in A and B, illustrated as blue and red boxes for Post-I and Exp swallows, respectively. Post-I Sw X bursts, however, partially overlapped despite a visible trough in the signal in between the early expiratory activity of this nerve (E1) and the swallow. When the vertical line between E1 and Sw is used to mark the start of the Post-I Sw X burst, it resulted in an artefactual decrease in the X burst duration and area under the curve (AUC). Graphical method developed to better estimate the start of the Post-I Sw X burst (**C**). The rising edge of the Sw X burst was used to draw a tangent line (oblique dotted line) down to the baseline (horizontal dotted line). The crossing point between the tangent line and the baseline was defined as the (estimated) start of the Post-I Sw X (black arrow), and this construct was used to compute the X burst AUC. Swallowing-related X and XII burst templates obtained in Exp Sw (red traces) were superimposed on those obtained during Post-I Sw for comparison purpose only. Note the similar burst duration and AUC between the swallow types.

2020 by the authors. Licensee MDPI. Basel. Switzerland. This article is an open access article distributed under the terms and conditions of the Creative Commons Attribution (CC BY) license (http://creativecommons.org/licenses/by/4.0/).
